# Feasibility of Using Unbound Mixed Recycled Aggregates from CDW over Expansive Clay Subgrade in Unpaved Rural Roads

**DOI:** 10.3390/ma9110931

**Published:** 2016-11-17

**Authors:** Isaac Del Rey, Jesús Ayuso, Adela P. Galvín, José R. Jiménez, Auxi Barbudo

**Affiliations:** Construction Engineering, University of Córdoba, Ed. Leonardo Da Vinci, Campus of Rabanales, Córdoba 14071, Spain; g52retii@uco.es (I.D.R.); g82pegaa@uco.es (A.P.G.); ir1jiroj@uco.es (J.R.J.); g82bamum@uco.es (A.B.)

**Keywords:** construction materials, mixed recycled aggregates, construction and demolition waste, expansive clays, unpaved rural roads.

## Abstract

Social awareness aims to increase practical skills, such as sustainable development, which seeks to increase the use of different types of waste in construction activities. Although insufficient attention is sometimes given to these actions, it is essential to spread information regarding new studies in the field of waste recycling, which encourages and promotes waste use. Reusing and recycling construction waste in the creation of buildings and infrastructure are fundamental strategies to achieving sustainability in the construction and engineering sectors. In this context, the concept of waste would no longer exist, as waste would become a material resource. Therefore, this study analyses the behaviours of two unbound mixed recycled aggregates (MRA) in the structural layers of an unpaved rural road with low traffic (category T43). The sections were built on inappropriate soil (A-7-6) with a high degree of free swelling. The experimental road consisted of three sections: the first was made with natural aggregates (NA) that were used as a control, the second was composed of MRA in the subbase and NA in the base, and the third section was completely composed of MRA. The materials were characterised in the laboratory. The behaviours of the structural layers in the experimental road were determined by controlling compaction (“in situ” density and moisture) and measuring the deflections and load capacity (deflectometer) during the 18 months after construction. The results show that the sections made with recycled aggregates meet the technical specifications required by General Technical Specifications for Road and Bridge Works (PG-3). Therefore, the water-soluble sulphate content and Los Angeles abrasion coefficient limits can be increased for recycled aggregates without compromising the quality of this type of road with low traffic. To the best of our knowledge, this is the first study regarding the use of unbound MRA made from construction and demolition waste (CDW) in the construction of an unpaved rural road with low traffic on an expansive clay subgrade.

## 1. Introduction

Waste management is an environmental, social and economic problem. Increasing consumption and the development of economic activities continue to generate large amounts of waste that require greater effort to reduce and prevent. In the past, waste was considered disposable and was disposed of in landfills. The current trends in waste management systems to replace removal with recycling and valorisation reflect the potential of waste as a resource rather than a problem. In 2012, the total amount of waste generated in the European Union (EU) by household activities amounted to 2514 million tons, of which 33% (821 million tons) was construction and demolition waste (CDW), which is the largest waste stream generated in the EU. During that same year, Spain generated a total waste of 119 million tons, of which 22% (26 million tons) was CDW [1].

The construction sector contributes significantly to environmental degradation, and it must move towards sustainability. Construction in developed countries accounts for 40% of global energy consumption over the use-life of materials (raw materials, construction, operations and dismantling) [2]. This results in a large contribution to greenhouse gas emissions, which contribute considerably to climate change [3]. In addition, the construction industry is responsible for 50% of the depletion of natural resources [4]. Therefore, reusing, recycling and revalorising CDW can conserve natural resources and reduce the volume of waste disposal in landfills.

The best practices for creating conditions that increase CDW recycling and improve the quality of recycling and recovery are being identified to present a set of recommendations to member states of the EU. It is necessary to address the potential barriers that maximise the generation of recycled aggregates (RA) from CDW. RA can be used in various civil engineering applications, such as in soil improvement projects and in the bases and subbases of roads. Additionally, CDW is used as a pipe bedding, backfilling and aggregate material for concrete and mortar.

Thus, RA are an excellent substitute for natural aggregate (NA) in civil engineering as unbound materials [5,6]. As many authors have shown [7,8,9,10,11], the mechanical properties of RA do not limit their use as unbound materials in roads. They can be used as base, subbase or embankment material. To allow a higher load distribution capacity in pavements, the use of RA in cement-treated granular materials (CTGM) provides excellent performance compared to using traditional unbound materials [12,13,14]. Additionally, RA can be used in recycled concrete [15,16,17] and mortar [18,19].

Although RA research has considerably progressed, the Spanish structural concrete code [20] only allows natural gravel to be replaced by recycled gravel, and RA can only be used in concrete (RCA). The code does not allow the use of mixed recycled aggregates (MRA) or ceramic recycled aggregates. Furthermore, recycled materials must meet the specifications of NA for the manufacturing of structural concrete. Not allowing the use of RA is a significant problem for CDW processing plants because most RA in Spain are MRA, representing over 80% of all CDW [21].

The Spanish General Technical Specification for Road and Bridge Works (PG-3) [22] using basic materials for pavements allows the use of RA, both MRA and RCA, as unbound materials, and CTGM can be used in structural layers and embankments. The use of MRA is vital in reducing landfill deposits. Experiments must be conducted to verify the performance of RA in the field. Jiménez et al. [8] performed an experiment with non-selected MRA (surface and base layers) on NA (subbase) and a subgrade classified as A-6 in accordance with the American Association of State Highway and Transportation Officials (AASHTO) [23]. Jiménez et al. [9] also conducted experiments using selected MRA (surface and base layers) on NA (subbase) and a subgrade classified as A-1-B in accordance with AASHTO. In both cases, the behaviour of the RA was similar to the behaviour of NA, and they found no significant differences between them.

The experimental road used in these studies is located in southern Spain (Andalusia). This rural area has abundant clay soils with high water-holding capacity, making them suitable for agriculture. Nevertheless, these clays are expansive and cause numerous structural failures in pavements [24].

Studies of the behaviour of recycled materials on expansive clay subgrades (inappropriate soil) have not been performed. Therefore, the response of RA over subgrades with high swelling capacity and low bearing capacity must be investigated.

The aim of this research is to study the behaviours of two MRA in the structural layers of an unpaved rural road with low traffic (category T43), i.e., less than 11 heavy vehicles per day, over a subgrade of expansive clays. The results are then compared to those when NA are used. To carry out this experiment, an 825 m experimental road was constructed with three different sections: one made with NA, another with MRA and a third section with both NA and MRA.

## 2. Materials and Methods

### 2.1. Design of the Experimental Unpaved Road Sections

**Figure 1:**
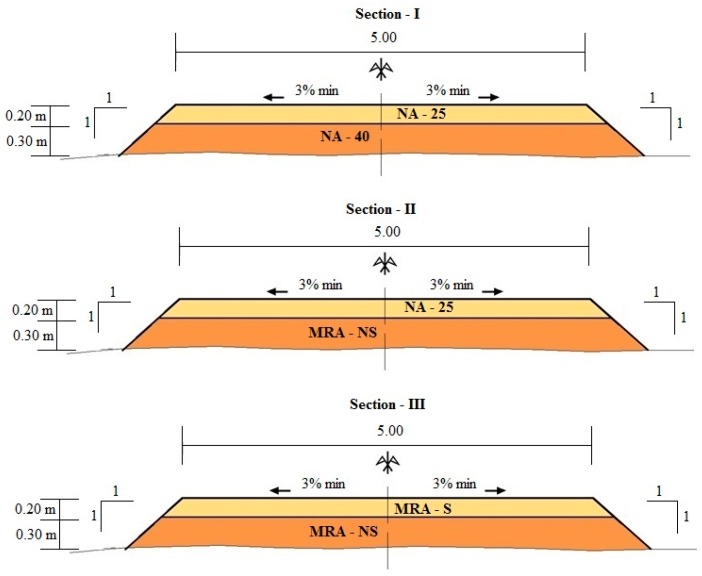
Illustration of the experimental sections of unpaved rural road.

The experimental road was constructed in southern Cordoba (Andalusia, Spain) in May 2014. It consists of a single carriageway with one lane five metres wide in both directions. The design of the structural layer was based on the bearing capacity of the subgrade and the estimated traffic density. The asphalt pavement manual for roads of low traffic intensity [25] was used to determine the thickness of the layers. Figure 1 shows the structural layers, cross slope, and the slope of the embankments of each section.

**Figure 2:**
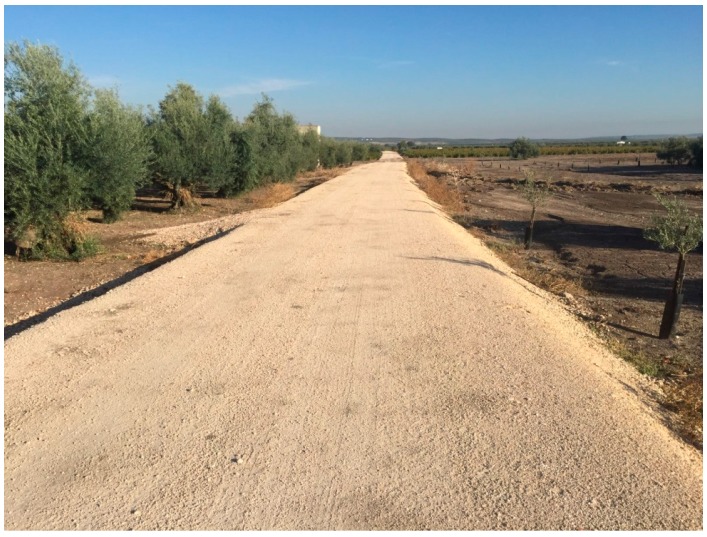
Section III of experimental unpaved rural road.

The experimental road was divided into three sections:

- Section I spans from KP (Kilometer Point) 0 + 0 to KP 0 + 275. Section I consists of two conventional materials; the subbase is an artificial graded aggregate with a 40 mm maximum size (NA-40), and the base is an artificial graded aggregate with a 25 mm maximum size (NA-25). This section will be used to compare the behaviours of recycled materials.
- Section II spans from KP 0 + 275 to KP 0 + 550. Section II consists of one conventional material and one non-selected recycled material. The aim of this section is to study the behaviour of a non-selected (NS), mixed recycled graded aggregate, with a 40 mm maximum size (MRA-NS) as the subbase layer. The base layer of this subsection is the same as that used in section I (NA-25).
- Section III (Figure 2) spans from KP 0 + 550 to KP 0 + 825. Section III consists of two recycled materials; the subbase is a non-selected, mixed, recycled, graded aggregate with a 40 mm maximum size (MRA-NS), the same as that used in Section II, and the base is a selected (S), mixed, recycled, graded aggregate with a 25 mm maximum size (MRA-S). The aim of this section is to study the behaviour of a structural layer built completely with recycled materials and to compare these behaviours with those of Section I, which uses conventional materials.

Structural layers of Section I were chosen as reference, Section III was carried out to evaluate the behaviour of MRA with respect to NA, and Section II was chosen to evaluate the behaviour of the subbase layer of MRA in case of finding structural failures in Section III.

### 2.2. Recycling Process of CDW

Two aggregates of mixed debris were used in the experimental sections, and the treatment process was different for each of them. MRA-NS was obtained without any pre-treatment. Mixed debris was crushed without any selection process to remove unwanted waste. By contrast, MRA-S is from selected waste. Furthermore, the mixed debris was pre-screened (20 mm) to remove unwanted impurities and topsoil (0–20 mm fraction), which can create plasticity and increase the sulphate content. After crushing, MRA-NS and MRA-S were screened to 40 mm and 25 mm, respectively. The aggregate retained by the sieve was transported to the crusher again for reduction.

In both recycled materials, metallic elements were removed using a magnetic conveyor belt. Lightweight materials were removed using a blower. Before the crushing process, a visual inspection was conducted to manually remove plastic, wood, paper, and other types of inappropriate materials.

### 2.3. Material Characterization

A complete characterisation of the materials that constitute the unpaved road sections of the subgrade, subbase, and base layers was performed. To perform the characterisation, 200 kg of each material was collected according to UNE-EN 932-1 [26]. The samples were taken during the construction of the experimental section before they were on-site. The tests performed were required under PG-3 [22]. Thorough reduction and sample preparation procedures were performed for each test in accordance with UNE-EN 932-2 standard [27].

#### 2.3.1. Subgrade Materials

**Figure 3:**
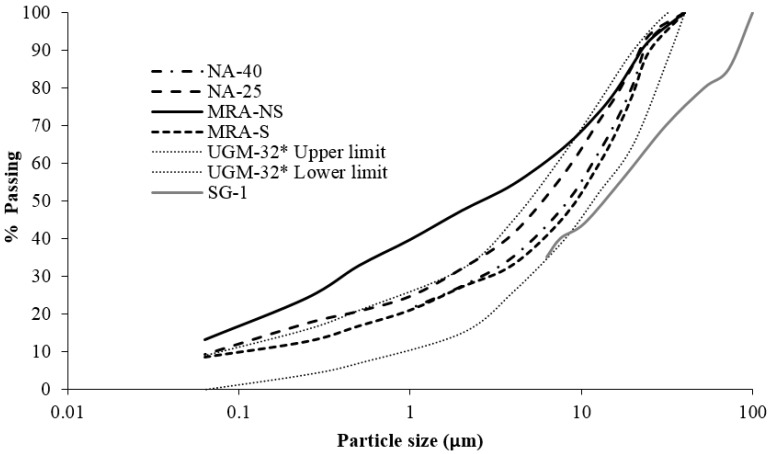
Particle size distribution. * UGM-32 = Unbound granular material. Maximum aggregate size is 32 mm.

The physical, mechanical and chemical properties of the subgrade were determined based on article 330 of PG-3 [22] which establishes the natural soil classifications and limit specifications used to formulate roadbeds and fills. The following factors were tested: the particle size distribution by sedimentation [28], plasticity index [29,30], the content of water-soluble sulphate [31], standard Proctor test (SPT) [32], California Bearing Ratio (CBR) [33], free swelling and collapse [34]. Table 1 shows the physical and mechanical properties studied. The particle size distribution is displayed in Figure 3.

The subgrade (SG-1) had a low bearing capacity, a CBR value less than 3, a low density, a high free swell (greater than 5%) and a collapse rate close to 2%. The organic matter content was below 1%. In accordance with the PG-3 [22], SG-1 was classified as inappropriate soil. According to the AASHTO [23], the soil is classified as A-7-6. In these soils, weather could produce shrinkage or swelling. This reduces the bearing capacity of the pavement and the failure of these type of roads by the action of traffic loads.

As can be seen in Figure 3, MRA-NS was outside the granulometric zones imposed by PG-3 [22]. MRA-NS, being a non-selected recycled waste material, had a higher percentage of fines required by the Spanish code.

#### 2.3.2. Subbase and Base Materials

The materials used in the experiments included two NA and two mixed MRA. The physical, mechanical and chemical properties of the NA and RA were determined based on article 510 of PG-3 [22], which regulates the properties and limits of materials used in the unbound structural layers of roads. The following factors were tested: the particle size distribution using a sieve [35], plasticity index [29,30], modified Proctor test (MPT) [36], the CBR [33], the flakiness index [37], the percentage of crushed particles [38], the sand equivalent [39], Los Angeles abrasion coefficient [40], and the contents of water-soluble sulphates and total sulphur [41]. Table 1 shows results of the physical and mechanical properties studied. The particle size distribution is displayed in Figure 3. The constituents of the RA were determined in accordance with UNE-EN 933-11 standard [42], and the results are shown in Table 2.

Similar to other studies, the CBR values obtained by RA are slightly lower than those obtained by NA [43,44]. L.A. abrasion coefficient of RA are above the limits of the PG-3 [22] (<35%) due to the high content of masonry waste [45]. Regarding the clean coefficient, all materials exceed the limit imposed by PG-3 [22] (<1%). These results are in accordance with findings by [10,46] in which the most limiting physical and mechanical properties of RAs are the L.A. coefficient and the clean coefficient.

The RA have higher contents of both water-soluble sulphate and total sulphur content compared to those of NA. MRA-NS has a total sulphur content that is higher than that allowed by PG-3 [22] (<1%); this occurs because the aggregate did not receive previous treatment to remove unwanted elements. In addition, the main source of sulphate in RA comes from gypsum [47]. Additionally, previous studies have found high levels of sulphate due to the presence of other CDW compounds such as mortar and ceramic particles [48,49,50].

### 2.4. Field Test and Quality Controls

#### 2.4.1. Field Density and Moisture Content

During road construction, the field density and moisture content were determined using nuclear density equipment according to ASTM D6938 [51]. One measurement was conducted every 25 m, i.e., 11 measurements were taken in each section. This test method is a quick and non-destructive technique for measuring the water content and dry density of the aggregate. The results were compared with the maximum dry density and optimum moisture values obtained from the modified Proctor test. The dry density and moisture content of the surface layer were measured at the completion of the experimental section (July 2014), after 12 months (July 2015) and after 18 months (January 2016).

#### 2.4.2. Plate Load Test

Static plate load tests (PLT) were used to determine the load-strain curves. The elastic modulus, which indicates the deformability characteristics of the soil, can be obtained from the slope of the secant through the points corresponding to 0.3 σ_max_ and 0.7 σ_max_, where σ_max_ is the maximum pressure applied. The load plate had a diameter of 300 mm, and the plate bearing test device had a load of 200 kN. One measurement of the subgrade and three measurements of the base and subbase layers were taken in each section in accordance with NLT-357/98 standard [52].

#### 2.4.3. Falling Weight Deflectometer

Tests were performed by applying a load and measuring the strain produced at the surface by the effect of the load. Deflection was measured using seven sensors (geophones). One sensor was positioned below the loading plate, and six were positioned at 0, 300, 450, 600, 900, 1200, and 1500 mm from the point of load application. The falling weight deflectometer (FWD, manufactured by Dynatest, Soborg, Denmark) was used to apply an impulse load to the road surface by dropping a steel bearing plate with a diameter of 450 mm. The drop height was adjusted to ensure a dynamic load of 40 kN. Two strokes were produced at each point. Measurements were made every 12.5 m, i.e., 22 measurements were taken in each layer in all the sections. The measurements were taken according to ASTM D4694 standard [53]. The deformation of the base layer was measured at the completion of the experimental section (July 2014), after 12 months (July 2015) and after 18 months (January 2016).

#### 2.4.4. Rut Depth Measurement

One of the most useful tests in assessing the possible failure or degradation of the pavement of a road is the measurement of rut depth on its surface. The permanent vertical deformations of the road surface along the unpaved rural road were registered following the standard of ASTM E1703/1703M [54]. This test was carried out in October 2016.

**Figure 4:**
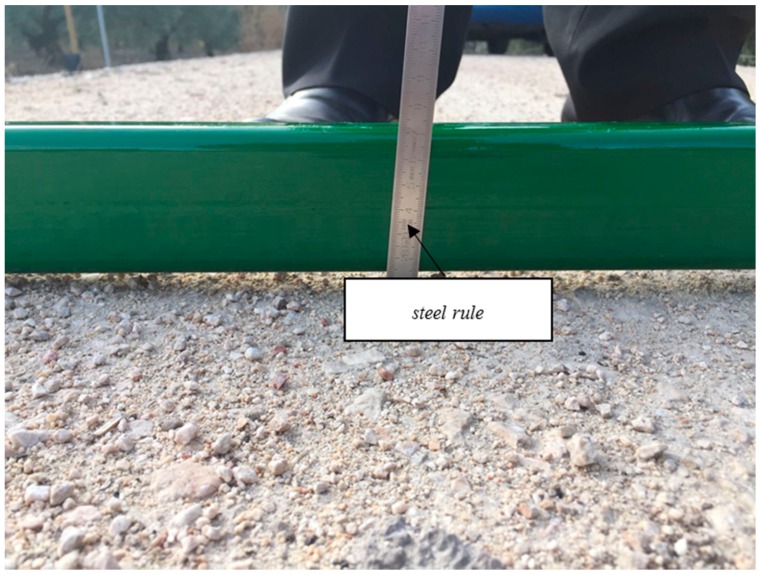
Rut depth measurement manually with straight edge and steel rule.

**Figure 5:**
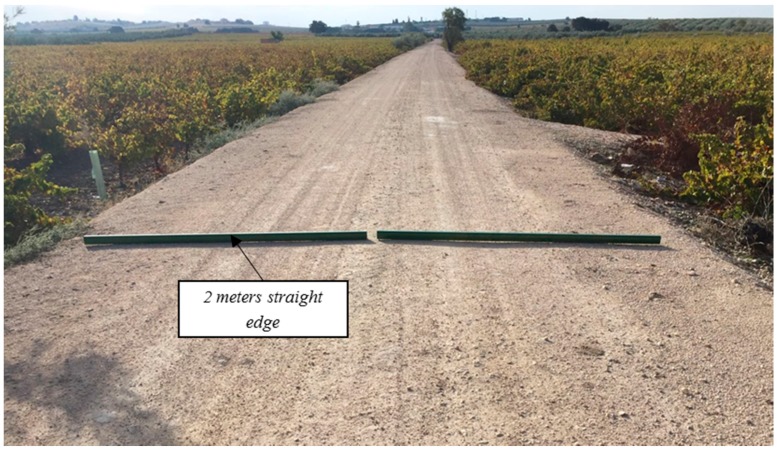
Straight edge of 2 m in each wheel path.

Assuming that the road surface in the middle has no rutting, the maximum rut depth in the left and right wheel path was measured manually with two straight edges of two meters long (Figure 4) and a steel rule calibrated with an accuracy of 0.5 mm (Figure 5). Measurements were performed every 30 m (10 transverse profiles in each of the three Sections).

## 3. Execution Quality Control and Experimental Behaviour

### 3.1. Field Quality Control of the Execution

In each section, quality control tests were carried out to assess the application of the materials in the subgrade, subbase, and base layers. Three “in situ” tests were conducted: dry density and moisture content tests using a Troxler nuclear gauge (manufactured by Troxler Electronics Lab., Durham, NC, USA), a bearing capacity test using a PLT, and deflection measurements using a FWD.

#### 3.1.1. Field Control of the Compaction

The degree of compaction is the most influential factor in the mechanical characteristics of the unbound material [55]. Therefore, the compaction process was controlled in the field. “In situ” moisture and density values were measured in the subgrade, subbase, and base. The degree of compaction was defined in accordance with the reference Proctor test. MPT was used for the pavement layers, and the SPT was used for the subgrade. SPT was used in the subgrade due to its advantage in expansive soils. PG-3 [22] indicates that the degree of compaction in the subgrade should meet SPT and MPT values of 95% and 98%, respectively, in pavement layers. The density and moisture values after compaction are shown in Table 3.

The compaction degree generally complied with PG-3 [22]. Hence, the experimental section was designed correctly. The average values in the subgrade sections were 97.7%, 96.4%, and 98.3% for sections I, II, and III, respectively. These results were higher than the 95% threshold required by PG-3 [22]. In the subbase sections, the average values were 99.8% in Section I, which used NA-40, and 100.2% and 99.9% in sections II and III, respectively, which used MRA-NS. In the base sections, the average values were 99.3% and 99.1% in sections I and II, respectively, which used NA-25, and 100.9% in Section III, which used MRA-S. In all the subbase and base layers, the results were higher than the 98% threshold required by PG-3 [22].

#### 3.1.2. Field Control of the Plate Load Test (PLT)

**Figure 6:**
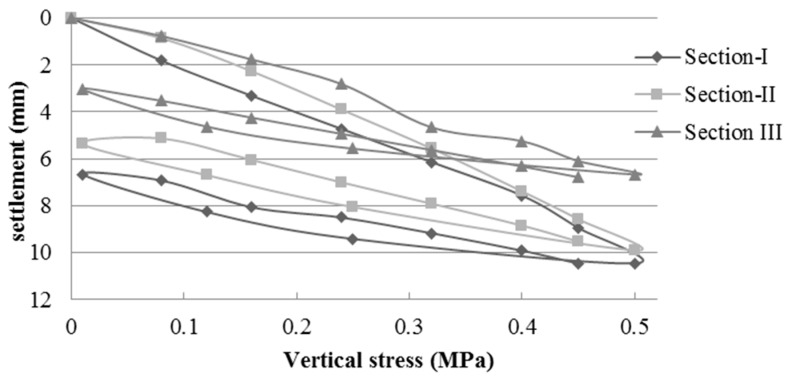
Vertical stress–settlement diagram of the subgrade layer.

**Figure 7:**
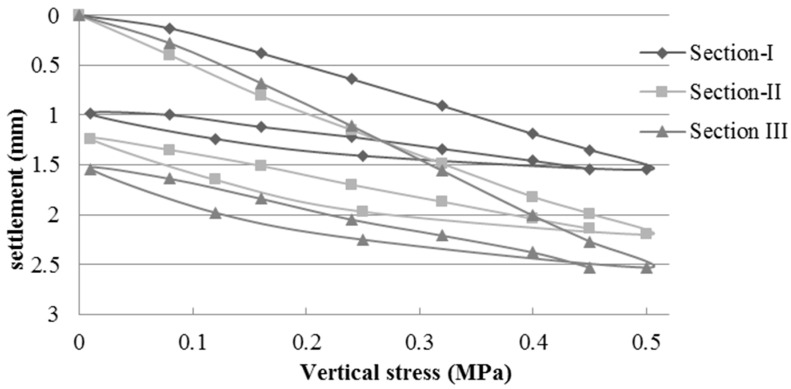
Vertical stress–settlement diagram of the subbase layer.

**Figure 8:**
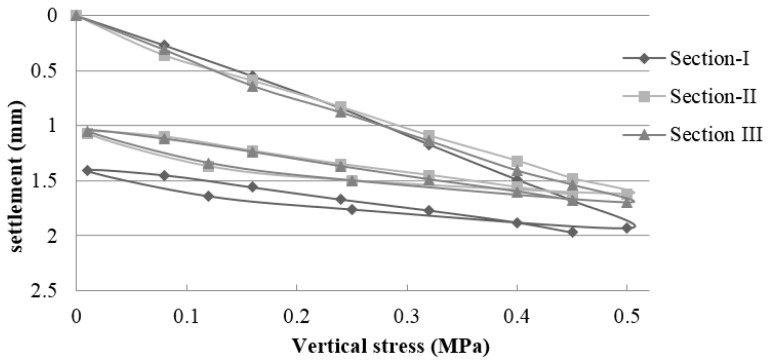
Vertical stress–settlement diagram of the base layer.

Based on the data obtained by the PLT, the elastic modulus was calculated for each load cycle. Table 4 shows the elastic modulus values in the second load cycle in each of the test sections according to NLT 357/98 standard [52]. Figure 6, Figure 7 and Figure 8 show the vertical stress-settlement diagrams of the subgrade, subbase, and base layers in each experimental section. The results are the average of two measurements per section.

The subgrade had an extremely low bearing capacity of less than 30 MPa, which is well below that required by the PG-3 [22] (≥100 MPa for selected soils and ≥60 for other soils). These values are consistent with a CBR that is less than 3 (Table 1). The bearing capacity was well below values obtained in previous studies that used soils with bearing capacity 10 times larger than those of the soils used in this study [8,9].

The elastic modulus values of the pavement are influenced by the subgrade properties. The low bearing capacity of the subgrade decreased the load capacity of the upper layers. The elastic modulus values of the subgrade ranged from 19.3 to 28.4 MPa. Compared to the subbase, the average value of Section I (162.0 MPa), which used NA, was 60% and 69% higher in relation to those of sections II (101.4 MPa) and III (95.9 MPa) respectively, which used MRA. In the base layer, the average value of Section I (170.1 MPa) was 6% greater than that of Section II (159.2) and 18% higher than that of Section III (144.7). The modulus of elasticity values of both NA and MRA were above the 80 MPa minimum, and the ratio of Ev2/Ev1 was less than the value of 2.2 required by PG-3 [22] for traffic category T43.

Jiménez et al. [8] constructed experiments using a subgrade with a modulus of elasticity greater than 300 MPa. Thus, the upper layers exhibited higher elastic modulus. The elastic modulus values of the subbase layers, which used MRA, were between 270 and 405 MPa, and those of the base layer varied between 370 MPa in the layer with RCA and 421 MPa in the layer with NA. As expected, the elastic moduli are higher than those obtained in similar experimental sections (I and II) in this study because they used an appropriate soil in the subgrade layer.

#### 3.1.3. Field Control of the Falling Weight Deflectometer

**Figure 9:**
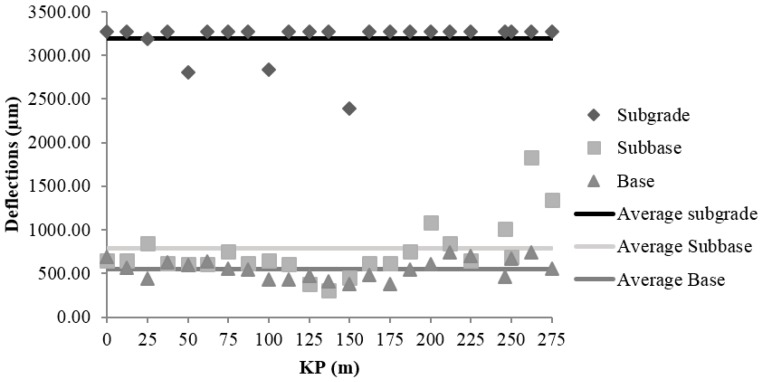
Deflections of subgrade, subbase and base in Section I.

**Figure 10:**
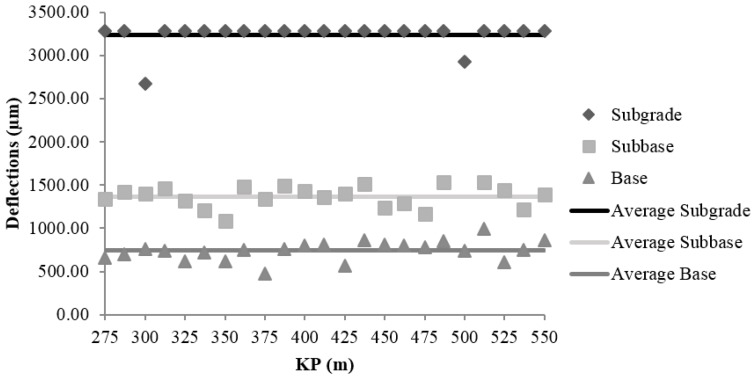
Deflections of subgrade, subbase and base in Section II.

**Figure 11:**
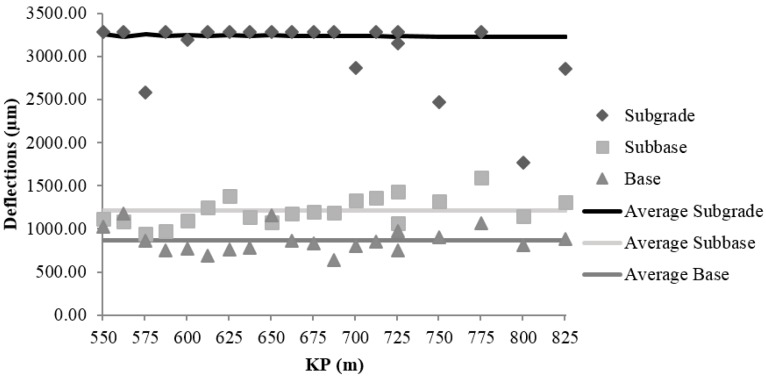
Deflections of subgrade, subbase and base in Section III.

The main FWD contribution is the analysis of the bearing capacity by reverse calculation of the modulus of elasticity values of the road layers based on the registered deflections. The deflections were measured with a Dynatest HWD 8081 deflectometer (manufactured by Dynatest, Soborg, Denmark). Figure 9, Figure 10 and Figure 11 show the values of deflection and the average deflection in the subgrade, subbase, and base in each of the sections.

The low bearing capacity of the subgrade is reflected by the high deflection values registered at the central geophone, which generally exceed 3000 µm. The average deflections in the subgrade were 3198 ± 37, 3219 ± 23, and 3073 ± 19 µm in sections I, II, and III, respectively.

The average deflections in the subbase layer were smaller relative to those in the subgrade: 74% in Section I (826 µm); 58% in Section II (1348 µm); and 61% in Section III (1209 µm). The values obtained in Sections II and III, which were constructed using MRA, are 63% and 46% higher than that in Section I respectively, which was made with NA. Nonetheless, the results in Sections II and III were admissible for unbound rural roads with low traffic and design speed, not compromising the structural stability of the road.

The decrease in the average deflections in the base layer, relative to those in the subbase, were 33% in Section I (552 µm), 45% in Section II (741 µm), and 32% in Section III (827 µm). In connection with the subgrade decrease were 82% in Section I, 77% smaller in Section II and 73% smaller in Section III. This result suggests that the behaviour of Section I, which was constructed with NA, is similar to the behaviour of Section III, which was constructed with MRA, and it demonstrates that the use of MRA does not compromise the load bearing capacity of the section.

Below the load application point, the equivalent elastic modulus (E) of the pavement structure at the centre of the plate (r = 0 mm) was calculated in each of the sections by applying the function proposed by Brown [56]:

$$E=\frac{2\left(1-\upsilon^{2}\right)\times \sigma \times r}{\delta }$$

where υ is the Poisson ratio of the material (0.35 for unbound granular materials in roads), σ is the stress applied below the plate, r is the plate radius (225 mm), and δ is the deflection at the centre of the plate measured in µm. The results of the elastic modulus are included in Table 5. These results were calculated using the average deflection in each section.

A decrease in the elastic modulus was observed in the section constructed with MRA, which may be related to the low resistance of the material to fragmentation [10,43,44,45,46]. In the subbase layer, sections II and III exhibited elastic modulus values that were 50.49% and 44.52% lower than those in Section I, which was constructed with NA. In the base layer, the modulus of elasticity in Section I was 15.68% higher than that in Section II and 27.09% higher than that in Section III. Although MRA undergo more deformation and have lower elastic modulus values than do NA, the MRA used in this study exhibited satisfactory performance.

### 3.2. Field Control of the Experimental Section Behaviour

An evaluation of MRA materials in an expansive clay subgrade under low-traffic conditions was conducted from July 2014 to January 2016. The main factors that affect the upper layers of unpaved roads with bases consisting of unbound granular materials are traffic density and weather conditions.

Table 6 shows the average monthly maximum and minimum temperatures and monthly total precipitation from execution until the completion of the performance tests. This information was collected from the nearest weather station, which is located in Cabra (Cordoba), with UTM coordinates of (373,516, 4,151,100). The elevation of the station is 547 m.

The temperatures were not extreme. Notably, 2015 was a particularly dry year. Precipitation was concentrated during the months of October to February.

As a service road to an agricultural area, the annual critical period is between September and February. During those months, increased vehicle traffic occurs, as this is the period of greatest agricultural activity. This period also coincides with the transition from a dry spell (summer) at the end of the period to maximum precipitation (winter). Therefore, the beginning of July 2015 and late January 2016 were chosen to perform “in situ” measurements of density, moisture, and falling weight deflection. These tests were carried out to observe the evolutional trends in the deformation, bearing capacity, and elastic modulus values.

#### 3.2.1. Field Control of Compaction Evolution

To evaluate the behaviour of compaction over time, three “in situ” moisture and density measurements were taken in each section: after execution, at 12 months and at 18 months. Each measurement was the average of 11 tests, and the results in each test section are included in Table 7.

A small decrease in the dry density over time was observed in sections I and II, whose bases were made with NA. Decreased dry density causes a decrease in the degree of compaction. The dry density in Section I decreased by 1.33% and 3.10% in July 2015 and January 2016, respectively. In Section II, the dry density decreased by 1.80% in July 2015 and 2.21% in January 2016. In Section III, a small increase in the dry density (0.53%) was observed in July 2015, and the dry density decreased by 1.07% in January 2016. As in previous investigations, the degree of compaction remained constant in the base layers constructed with recycled aggregates [8], although minimal loss of compaction was observed in the sections constructed with NAs in these studies.

As expected, RA exhibited a higher moisture content than did NA. The high porosity and absorption of MRA increased the moisture content (Table 1). This difference was largest after a large precipitation event in January 2016 (88.1 mm).

To assess the significance of the effect of the two factors (composition of each section and date) have on dry density, an analysis of variance (ANOVA) was conducted with the statistical software Statgraphics Centurion XVI (Version 16.1.18, Statgraphics, Madrid, Spain). The F-test in the ANOVA analysis was used to evaluate whether one factor had statistically significant effects on dry density, with a 95% confidence level. If the p-value was lower than 0.05, the factor showed a significant effect on the property studied. To check whether there was a significant difference between the levels for each factor, Fisher’s Least Significant Difference (LSD) test was conducted. In this method there are statistically significant differences at a 95% confidence level when there are non-overlapping bars.

**Figure 12:**
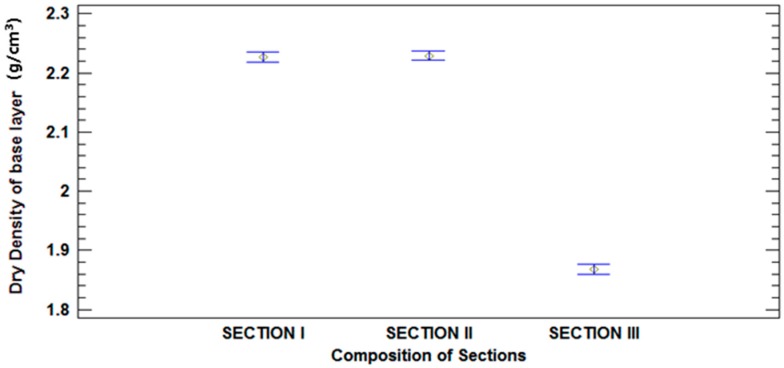
Mean values of dry density and 95% LSD intervals vs. composition of sections.

**Figure 13:**
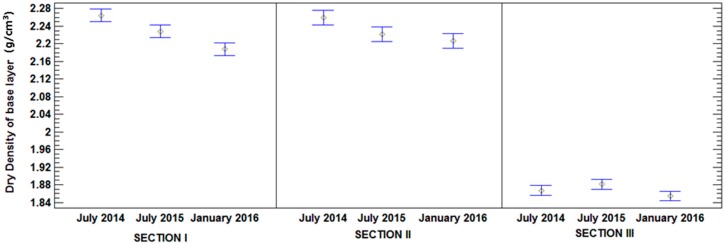
Mean values of dry density and 95% LSD intervals vs. date.

Table 8 shows the results obtained with the ANOVA. The results indicate that the composition of sections has a statistically significant influence on dry density (*p* < 0.05). In relation to date, the results show that this factor has statistically significant influence (*p* < 0.05) on Section I and Section II. However, there is no influence in Section III. Figure 12 and Figure 13 illustrate the average and 95% LSD Interval. No statistically significant differences were found between those levels that share overlapping bars. The least significant difference (LSD) method was used for this analysis. In this method, 5% is associated with saying that every pair of means is significantly different when the actual difference is equal to 0.

As expected, the mean values of Section III, the base layer was performed with MRA, were lower than the mean values of Section I and II, constructed with NA. There were statistically significant differences at a 95% confidence level between Section I and II compared to Section III, as is indicated by the non-overlapping bars in Figure 10.

As can be seen in Figure 13, according to overlapping of bars, Section III did not exhibit a decrease in dry density, there were no statistically significant differences in densities measured over time. However, sections I and II displayed statistically significant differences in their dry density values, which decreased over time. Density loss was mainly due to the base material. Both sections were made with NAs and were differentiated by the subbase materials. Thus, no significant difference was observed between the behaviours of sections I and II. Section I was made completely with NA, while the subbase of Section II was made with MRA. These results could be due to the higher absorption capacity of MRA (Table 1), which alleviates the effects of precipitation and humidity.

Similar results were obtained by other authors. In sections with RA base layers, Jiménez et al. [8] found no statistically significant differences over the first two years; however, heavy traffic produced a dry density increase of 4% during the third year. The same results were obtained by Jiménez et al. [9] in sections made with an NA base layer. Although they observed no statistically significant differences, a small decrease in the dry density, approximately 2%, was observed. A similar decrease in the degree of compaction was observed in this study. Based on these results, the degree of compaction of the base layer made with RA exhibited a minor decrease compared to that of the base layer made with NA.

#### 3.2.2. Field Control of the Evolution of the Deflection and Bearing Capacity

**Figure 14:**
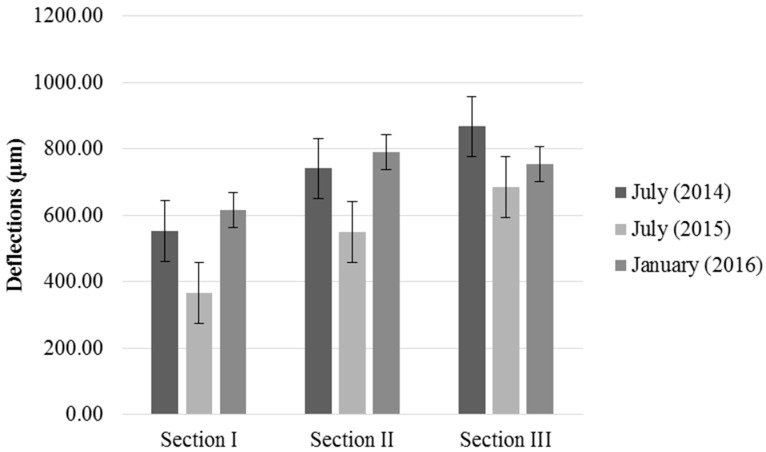
Evolution of the deflection for the base layer over time.

The behaviour of the base layer was analysed based on measurements collected using the FWD. The bearing capacity was analysed using reverse calculations of the modulus of rigidity of the road layers based on the registered deflections. Figure 14 shows the evolution of deflection over time. The values are the average of 22 measurements per section.

The evolution of deflection was similar in Section I and Section II, the deflection decreases in July 2015 and increases in January 2016, the deflections in January 2016 were higher than in July 2014. In Section III, the deflection decreases in July 2015 and increases in January 2016, but does not outnumber January 2016. The deflections were lower in July 2015 than in July 2014, which had dry conditions and low soil moisture. The average deflections observed in sections I (336.51 µm) and II (549.81 µm) were lower than those observed in Section III (685.41 µm). Reductions of 33.65%, 25.81%, and 20.98% compared to July 2014 were observed in sections I, II, and III, respectively. After a large precipitation event in January 2016, high deflection was observed in all the sections. The average deflections were 616.13, 789.59, and 753.41 µm in sections I, II and III, respectively. In sections with base layers made of NA (Section I and II), the deflection at 18 months was greater than that obtained after completion of the section, as it increased by 11.61% in Section I and 6.54% in Section II. Although an increase in deflection was observed in Section III compared to previous measurements, the deflection at 18 months (January 2016) was 13.14% lower than that observed after completion of the section (July 2014).

To assess the effect of composition of section and date on deflections a similar ANOVA was performed. Table 9 presents a summary of the results. No statistically significant differences were found between those levels that share overlapping bars.

**Figure 15:**
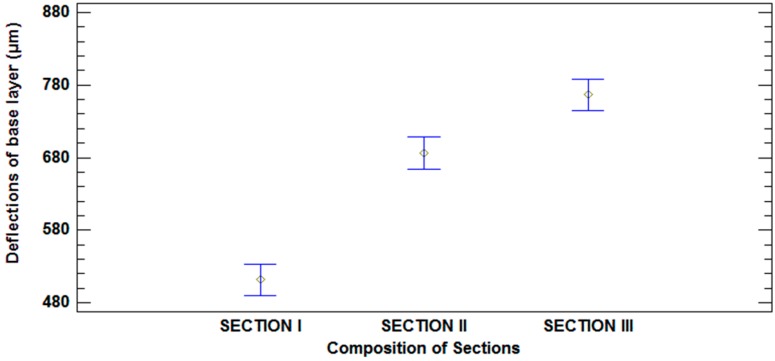
Mean values of deflections and 95% LSD intervals vs. composition of sections.

There were statistically significant differences at a 95% confidence level, as can be seen in Figure 15.

**Figure 16:**
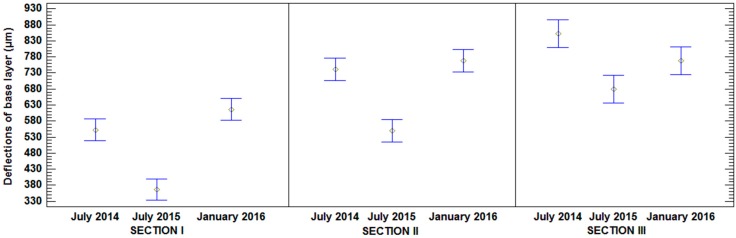
Mean values of deflections and 95% LSD intervals vs. composition of sections.

According to overlapping of bars in Figure 16, there were significant differences in deflections measured over time in all sections, although it is appreciated that the tendency of deflections was different between them. Section I and II showed an increase of deflections with the onset of rains and, therefore, increased humidity. Deflections in January 2016 were higher than deflections in July 2014, after the execution of the road. However, this did not happen in Section III, in which the deflections increased in January 2016 compared to July 2015, but did not reach the deflections of July 2014. As in dry density, these results are due to the higher absorption capacity of MRA (Table 1), which alleviates the effects of precipitation and humidity.

**Figure 17:**
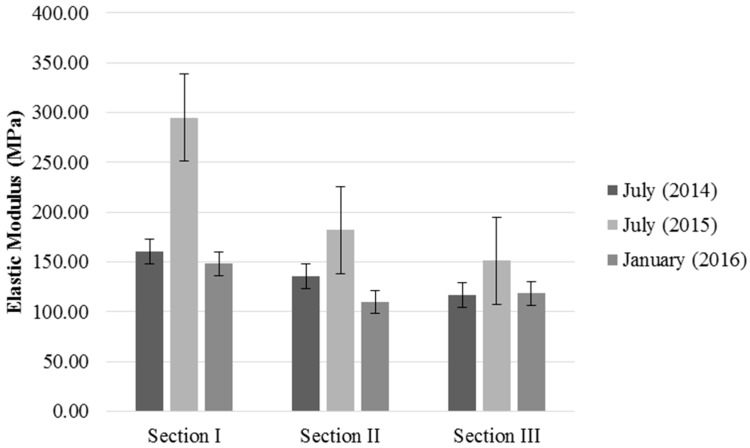
Evolution of the elastic modulus for the base layer over time.

The elastic modulus was calculated from the deflections [56]. Figure 17 shows the evolution of the modulus of elasticity over time.

All of the elastic modulus values complied with the requirements of PG-3 [22] (>80 MPa). In Section I, the elastic modulus decreased to 148.2 MPa in January 2016, a reduction of 7.62% from July 2014. In Section II, the elastic modulus was 109.9 MPa in January 2016, a decrease of 18.72% from July 2014. In Section III, the elastic modulus was 118.2 MPa in January 2016, a decrease of 1.04% compared to July 2014. The increase in the elastic modulus in Section III is due to the pozzolanic activity or hydraulic properties of the RA cement [10,57,58].

These results are consistent with the moisture data shown in Table 7. In July 2015, the soil moisture content was low, and deflections were small; therefore, the material behaved more rigidly. However, deflection increased and the modulus of elasticity decreased when the moisture content increased in January 2016.

### 3.3. Field Control of the Rut Depth

The permanent vertical deformation of the road surface is the main failure mechanism for unpaved roads. Rutting is due to permanent deformations in any of a pavement’s layers or subgrade usually caused by consolidation or lateral movement of the materials due to traffic loading. The combination of these actions, coupled with wet weather conditions, increase the vertical deformations of the road surface resulting in severe rut depth and potholes [59,60]. Once the wheel path is too deep, driving can be dangerous due to the instability of the surface layer and its rutting will ultimately leave the road with permanent deterioration, which leads to the requirement to reshape the entire cross section [59].

Table 10 shows the measurement of the rut depth to the left and right wheel path for each section.

The AASHTO Guide for Design of Pavement Structures [61] suggests that the allowable rut depth for unpaved roads is between 25.4 mm and 50.8 mm (1 and 2 inches). As seen in Table 10, in any of the positions tested these values have not been exceeded. All values are less than 20 mm, with the average close to 10 mm. With regard to Section I, with an average of 10.3 mm, the rut depth in Section II increased by 6.5% and Section III decreased by 6.6%.

The Guideline of Pavement Surface Condition Rating Manual [62] suggests that there is a low level of severity for a rut depth of less than 10 mm, a medium level of severity for rut depth between 10-20 mm, and high severity level for rut depth greater than 20 mm. Considering that these values are for paved roads, the unpaved road has performed correctly, since Section III was below 10 mm of rut depth and Sections I and II presented rutting close to 10 mm.

**Figure 18:**
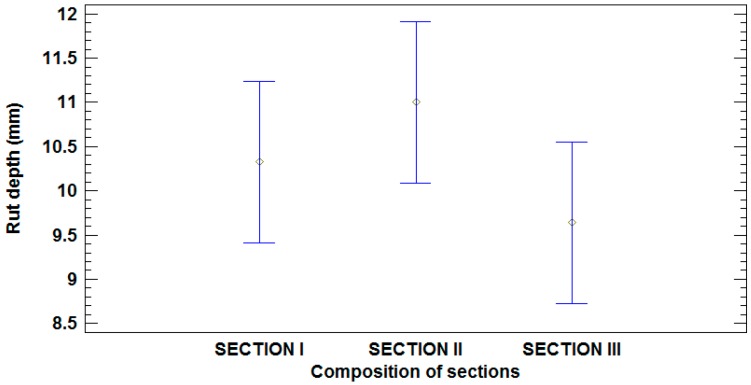
Mean values of rut depth and 95% LSD intervals vs. composition of sections.

To assess the composition effect of sections on rut depth, a similar ANOVA was performed. Table 11 presents a summary of these results, finding statistically non-significant differences between those levels that share overlapping bars (Figure 18).

## 4. Conclusions

In this study, the behaviours of two MRA of CDW were evaluated under field conditions. A low-traffic unpaved road was built on an expansive clay subgrade. This material was classified as inappropriate according to PG-3 due to its high free swelling (over 5%). Additionally, it was classified as A-7-6 according to the AASHTO. The following conclusions were drawn from this study:

- Section III, which was constructed with MRA, did not exhibit a decrease in the dry density, and there were no significant differences in the densities measured over time. However, sections I and II exhibited significant differences in their dry densities, including decreased density over time. Density loss was mainly due to the base material. Because both sections were made with NA, no significant difference was found between the behaviours of sections I and II, which were differentiated by the subbase material type. Section I was constructed with NA, while the subbase of Section II was constructed with MRA.
- Deflections were measured over 18 months. In all of the measurements, the deflections in the section made with NA, both the base and subbase, were lower than those in sections that used MRA in some structural layers. In July 2014 and July 2015, the deflections in Section II, whose subbase was made with MRA, were lower than those in Section III, which was constructed with MRA in the base and subbase. In January, the deflections in Section III were lower than those in Section II. In the base layer made of MRA, a low degree of compaction resulted in smaller deflections. Deflections in sections with MRA in January 2016 were less than 800 (µm) which is considered acceptable for this type of road.
- The elasticity modulus of the base layers of the three sections, which were measured using an FWD, satisfied the requirements for unpaved rural road, this value exceeded 100 MPa in the three sections.
- Permanent vertical deformations measured in the wheel paths, after more than two years since the opening of the road, were lower or very close to 10 mm. Therefore, the severity level of rut depth was low. The lowest values were obtained by Section three, which was performed only with recycled materials
- The results of this study suggest that MRA can be used in structural layers of unpaved roads with low traffic over subgrades of expansive clays. In addition, the L.A. abrasion coefficient and total sulphur content requirements established by the Spanish Technical Specification could be increased for these roads.

Thus, according to the experimental data obtained in this study, we recommend the use of RA in unpaved rural roads with low traffic. The use of RA could reduce the volumes of construction and demolition waste in landfills and increase the recycling rate. Consequently, the commercial values of these recycled materials would increase while achieving environmentally sustainable development in the construction and engineering sectors.

## Figures and Tables

**Table 1:** Physical, mechanical and chemical properties.

Properties	Particle Size	SG-1	NA-40	NA-25	MRA-NS	MRA-S
Water absorption (%)	>4 mm	–	4.3	3.8	8.9	9.4
	<4 mm	–	3.8	3.7	7	7.2
Density-SSD (g/cm^3^)	>4 mm	–	2.423	2.393	2.11	2.18
	<4 mm	–	2.467	2.471	2.25	2.24
Liquid limit (LL)	–	52.7	–	–	–	–
Plastic limit (PL)	–	29.3	–	–	–	–
Plasticity index (PI)	–	23.4	No	No	No	No
Sand equivalent (%)	–	–	35	37	45	68
Clean coefficient (%)	–	–	2.7	1.4	8.7	7.3
Los Angeles (L.A.) Coefficient (%)	–	–	30.3	31.6	47	40
Flakiness index (%)	–	–	10.5	13.5	24	12.8
Crushed particles (%)	–	–	100	100	91	97
Maximum dry density (MPT) (g/cm^3^)	–	–	2.29	2.28	1.91	1.85
Maximum dry density (SPT) (g/cm^3^)	–	1.52	–	–	–	–
Optimum moisture content (%)	–	27.3	7.4	6.4	11.4	11.6
CBR (%)	–	2.7	48.9	78.4	67.3	63.7
Free swelling (%)	–	6.96	–	–	–	–
Collapse (%)	–	1.93	–	–	–	–
Organic matter (%)	–	0.74	0	0	0.02	0.01
Water soluble sulphate (% SO_4_)	–	0.01	0.01	0.01	0.27	0.22
Total sulphur content (% S)	–	0.27	0.02	0.01	1.15	0.65

**Table 2:** Compositions of coarse recycled aggregates.

Compositions	MRA-NS	MRA-S
% Ra (Asphalt)	0.9	1.6
% Rb (Ceramic)	28.9	33.7
% Rc (Concrete and mortar)	45.7	40.7
% Ru (Natural aggregate)	22.4	23.2
% Rg (Glass)	0.0	0.0
FL (Floating particles) (cm^3^/kg)	2.4	1.0
% X1 (Gypsum)	0.8	0.3
% X2 (Wood, plastic and metals)	0.9	0.4

**Table 3:** “In situ” assessments of density and humidity.

KP	Subgrade			Subbase			Base		
	Dry Density (g/cm^3^)	Moisture (%)	Compaction (% SPT)	Dry Density (g/cm^3^)	Moisture (%)	Compaction (% MPT)	Dry Density (g/cm^3^)	Moisture (%)	Compaction (% MPT)
0 + 12.5	1.48	27.6	97.4	2.26	4.7	98.7	2.26	3.4	99.1
0 + 37.5	1.51	24.3	99.3	2.27	4.7	99.1	2.27	2.6	99.6
0 + 62.5	1.50	26.6	98.7	2.25	3.4	98.3	2.30	2.6	100.9
0 + 87.5	1.43	17.1	94.1	2.36	4.0	103.1	2.27	2.5	99.6
0 + 112.5	1.46	28.2	96.1	2.30	3.2	100.4	2.28	4.1	100.0
0 + 137.5	1.47	22.7	96.7	2.26	4.0	98.7	2.25	3.8	98.7
0 + 162.5	1.58	20.8	103.9	2.26	3.8	98.7	2.25	2.5	98.7
0 + 187.5	1.55	18.0	102.0	2.30	3.2	100.4	2.26	2.4	99.1
0 + 212.5	1.44	26.8	94.7	2.36	4.0	103.1	2.23	2.6	97.8
0 + 237.5	1.42	23.3	93.4	2.26	4.1	98.7	2.25	3.3	98.7
0 + 262.5	1.49	25.6	98.0	2.26	3.8	98.7	2.29	3.3	100.4
Average	1.48 ± 0.05	23.73 ± 3.78	97.7 ± 3.2	2.29 ± 0.04	3.90 ± 0.51	99.8 ± 1.8	2.26 ± 0.02	3.01 ± 0.59	99.3 ± 0.9
0 + 287.5	1.41	29.6	92.8	2.02	7.1	105.8	2.25	3.7	98.7
0 + 312.5	1.55	23.4	102.0	1.93	9.6	101.1	2.27	2.9	99.6
0 + 337.5	1.51	22.5	99.3	1.94	9.4	101.6	2.25	3.6	98.7
0 + 362.5	1.51	22.5	99.3	1.91	11.1	100.0	2.22	3.7	97.4
0 + 387.5	1.48	23.0	97.4	1.93	10.2	101.1	2.25	3.6	98.7
0 + 412.5	1.44	25.0	94.7	1.86	8.2	97.4	2.24	3.3	98.3
0 + 437.5	1.48	18.7	97.4	1.87	8.2	97.9	2.23	3.4	97.8
0 + 462.5	1.42	28.3	93.4	1.93	9.6	101.1	2.27	3.9	99.6
0 + 487.5	1.43	17.8	94.1	1.87	8.2	97.9	2.23	3.7	97.8
0 + 512.5	1.42	28.1	93.4	1.94	9.7	101.6	2.25	3.2	98.7
0 + 537.5	1.47	20.9	96.7	1.86	9.8	97.4	2.40	3.6	105.3
Average	1.47 ± 0.05	23.62 ± 3.85	96.4 ± 3.0	1.91 ± 0.05	9.19 ± 1.14	100.2 ± 2.5	2.26 ± 0.05	3.51 ± 0.28	99.1 ± 2.2
0 + 562.5	1.45	23.8	95.4	1.90	9.8	99.5	1.91	9.7	103.2
0 + 587.5	1.48	19.3	97.4	1.91	10.1	100.0	1.90	9.6	102.7
0 + 612.5	1.52	23.5	100.0	1.91	8.4	100.0	1.89	11.7	102.2
0 + 637.5	1.45	21.7	95.4	1.92	10.7	100.5	1.85	12.2	100.0
0 + 662.5	1.51	23.3	99.3	1.86	8.8	97.4	1.82	11.4	98.4
0 + 687.5	1.44	17.8	94.7	1.94	10.6	101.6	1.82	10.7	98.4
0 + 712.5	1.46	25.2	96.1	1.89	9.5	98.9	1.88	11.5	101.6
0 + 737.5	1.42	27.7	93.4	1.95	8.4	102.1	1.85	11.7	100.0
0 + 762.5	1.47	19.9	96.7	1.89	11.0	98.9	1.89	11.5	102.2
0 + 787.5	1.62	15.3	106.6	1.90	11.1	99.5	1.91	10.4	103.2
0 + 812.5	1.61	17.3	105.9	1.92	10.9	100.5	1.82	10.7	98.4
Average	1.49 ± 0.07	21.35 ± 3.76	95.3 ± 4.4	1.91 ± 0.02	9.94 ± 1.03	99.9 ± 1.3	1.87 ± 0.04	11.01 ± 0.85	100.9 ± 2.0

**Table 4:** Elastic modulus in MPa.

Section	Subgrade			Subbase			Base		
	Ev_1_	Ev_2_	Ev_2_/Ev_1_	Ev_1_	Ev_2_	Ev_2_/Ev_1_	Ev_1_	Ev_2_	Ev_2_/Ev_1_
Section I	13.7	28.4	2.1	77.5	162.0	2.1	83.1	170.1	2.1
Section II	10.8	19.3	1.2	52.6	101.4	1.9	73.2	159.2	2.2
Section III	13.9	25.9	1.9	45.9	95.9	2.1	70.0	144.7	2.1

**Table 5:** Elastic modulus of the pavement structure in MPa.

Section	Subgrade	Subbase	Base
Section I	26.8	147.1	160.4
Section II	29.1	72.8	135.2
Section III	30.9	81.6	116.9

**Table 6:** Temperatures and precipitation from execution until completion.

Month	Minimum Temperature (°C)			Maximum Temperature (°C)			Precipitation (mm)		
	2014	2015	2016	2014	2015	2016	2014	2015	2016
January	–	4.43	6.88	–	13.71	13.85	–	49	88.1
February	–	2.88	6.52	–	11.63	14.3	–	55.8	145
March	–	7.03	–	–	18.50	–	–	39.6	–
April	–	10.09	–	–	21.78	–	–	41	–
May	–	14.36	–	–	28.71	–	–	0.6	–
June	–	16.33	–	–	31.08	–	–	7.8	–
July	17.42	22.31	–	31.32	36.81	–	0	0	–
August	18.00	18.37	–	32.10	31.83	–	0	2.3	–
September	15.80	15.19	–	27.22	27.30	–	6.2	25.5	–
October	14.97	13.53	–	25.37	22.56	–	50.2	78.3	–
November	9.09	9.44	–	16.54	19.49	–	130	44.8	–
December	4.98	9.50	–	13.20	18.50	–	21.4	2.2	–
Average	11.34	11.96	6.70	22.07	23.49	14.08	–	–	–
Total	–	–	–	–	–	–	207.8	346.9	233.1

**Table 7:** Average values of dry density, moisture and compaction.

Date	Properties	Section I	Section II	Section III
July 2014	Dry Density (g/cm^3^)	2.26	2.26	1.87
	Moisture (%)	3.01	3.51	11.01
	% Compaction	99.3	99.1	100.9
July 2015	Dry Density (g/cm^3^)	2.23	2.22	1.88
	Moisture (%)	2.07	2.25	5.13
	% Compaction	97.7	97.5	101.7
	Dry Density (g/cm^3^)	2.19	2.21	1.85
January 2016	Moisture (%)	5.33	4.55	10.58
	% Compaction	96.0	96.8	100.1

**Table 8:** Dry density results of ANOVA and coefficient of variation.

Properties	Factor Levels	Factor											
		Composition of Sections			Date								
		Section I	Section II	Section III	Section I			Section II			Section III		
					July 2014	July 2015	January 2016	July 2014	July 2015	January 2016	July 2014	July 2015	January 2016
Dry Density (g/cm^3^)	*p*-value	<0.0001			<0.0001			0.0073			0.0554		
	Average	2.23	2.23	1.87	2.26	2.23	2.19	2.26	2.22	2.21	1.87	1.88	1.85
	c.v.	2.02	1.94	1.40	0.89	1.91	1.47	2.17	0.98	1.72	1.95	0.90	0.74

c.v. = Coefficient of variation (%); bold *p*-values show significant difference.

**Table 9:** Deflection results of ANOVA and coefficient of variation.

Properties	Factor Levels	Factor											
		Composition of Sections			Date								
		Section I	Section II	Section III	Section I			Section II			Section III		
					July 2014	July 2015	January 2016	July 2014	July 2015	January 2016	July 2014	July 2015	January 2016
Deflections (µm)	*p*-value	<0.0001			<0.0001			<0.0001			0.0008		
	Average	511.667	686.164	766.553	552.36	366.51	616.13	741.12	549.81	789.59	867.38	685.41	753.41
	c.v.	0.04	0.02	0.03	20.14	33.84	20.39	15.24	19.64	12.25	17.07	25.29	20.26

c.v. = Coefficient of variation (%); bold *p*-values show significant difference.

**Table 10:** Rut depth measured by section in millimetres (mm).

Point	Section I		Section II		Section III	
	Left	Right	Left	Right	Left	Right
P1	13.5	16.0	16.0	17.0	10.0	11.5
P2	7.5	6.5	6.0	11.0	5.0	8.0
P3	8.5	10.0	9.5	13.0	8.0	9.5
P4	8.5	10.0	9.0	13.5	8.0	9.0
P5	13.5	9.5	9.5	10.5	10.5	10.0
P6	11.0	8.5	10.0	10.0	5.0	13.5
P7	17.5	13.5	11.0	13.5	8.0	14.0
P8	9.0	9.0	10.5	9.5	10.0	11.5
P9	5.0	6.5	12.0	9.0	10.0	6.5
P10	13.5	9.5	10.0	9.5	8.8	16.0
Average by wheel path	10.8 ± 3.7	9.9 ± 2.9	10.4 ± 2.5	11.7 ± 2.5	8.3 ± 2.0	11.0 ± 2.9
Average by section	10.3 ± 3.3		11.0 ± 2.6		9.6 ± 2.8	

**Table 11:** Rut depth results of ANOVA and coefficient of variation.

Properties	Factor			
	Factor Levels	Composition of Sections		
		Section I	Section II	Section III
Deflections (mm)	*p*-value	0.3378		
	Average	10.3	11.0	9.6
	c.v.	0.32	0.23	0.29

c.v. = Coefficient of variation (%).
